# Facts about Steel

**Published:** 1897-09

**Authors:** 


ARTICLE III.

### FACTS ABOUT STEEL.

A prominent manufacturer has issued what he calls a steel catechism, giving much information about steel. It is as follows:

Q. What is steel?

A. A metal composed of from 97 per cent to 99.06 per cent. of iron, and from 3 per cent. to 0.04 per cent. of carbon is properly called steel. Many other substances, however, are commonly found in steel. Among these are sulphur, phosphorus, silicon, copper and manganese. These ingredients give various properties to the metal according to the amounts present. There are also several alloys of steel with other metals, but these are generally designated by hyphenated titles, such as "chromo-steel," "nickel-steel," etc.

Q. How is steel made?

A. In many ways, if we consider details. The principal methods are three. The resulting products are known as crucible steel, Bessemer steel, and open hearth steel.

Q. What is crucible steel?

A. Crucible steel is obtained as the result of fusing together in a crucible the substances desired in any quantity of steel.

Q. What is Bessemer steel ?

A. Bessemer steel is made by forcing a blast of air through melted iron. By this process the substances not desired in the steel are burned up. As some ingredients which are needed are also consumed, these substances (carbon, etc.,) are added before the liquid metal is permitted to cool.

Q. What is open hearth steel ?

A. Chemically the open hearth method is substantially the same as the Bessemer process. But in making open hearth steel the forced blast is done away with and the metal while melting and after complete liquefaction is kept exposed to the air in such a way that the impurities are oxidized or slowly burned out of the iron. The constituents other than iron are added before solidification. It is wholly immaterial whether the mixing of the iron, carbon, manganese and the rest is done in a crucible, a Bessemer converter, or an open hearth furnace, so long as the same materials are compounded with equal skill, and the steels treated after manufacture with the same care and judgment.

Q. What is "cast steel ?"

A. All steel made by the processes which we have discussed is really "cast steel." That is, the metal becomes steel while melted, and is then run in molds. The name, "cast steel" should be restricted to steel which is cast in the shape in which it is intended to be used. The result is inferior for most purposes to that obtained by forging and similar methods. There is much confusion, however, in the popular use of this term.

Q. What is "tool steel ?"

A. The term has no scientific meaning. Those who use it mean any steel suitable for making tools. It may be crucible steel, Bessemer steel, open hearth steel, or steel made by some less important method. For no good reason the name, "tool steel," is often regarded with almost superstitious awe. The agent of the steel-maker calls a substance "tool steel," knowing little or nothing of its composition and properties. The steel-user buys it, the workman makes things of it, the consumer buys and uses the things. Sometimes the steel is adapted to its use and everybody is satisfied. Sometimes it is not, and the consumer finds fault and the agent sells no more steel to that manufacturer. Meantime nobody concerned, except the original maker, has the least idea what the stuff called "tool steel" really is. No manufacturer knows what he is buying unless he has a laboratory where his purchases can be tested.

Q. What is "machine steel."

A. That is another shop name. It is applied to any steel which is soft enough to be easily cut and drilled.

Q. By the way, what is nickel-steel ?

A. A steel containing, besides the ordinary ingredients, perhaps 5 per cent. of nickel. It has a tensile strength of over 100,000 pounds to the square inch against about 55,000 pounds in the tubing ordinarily used.

